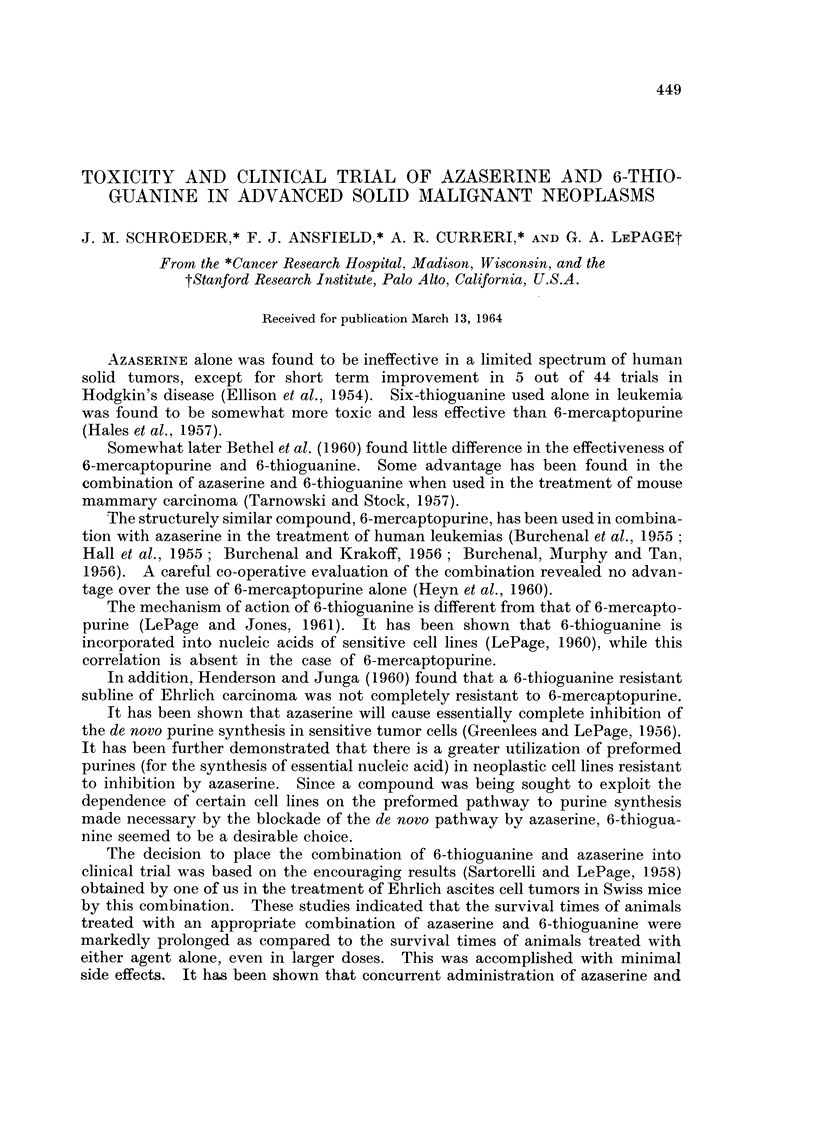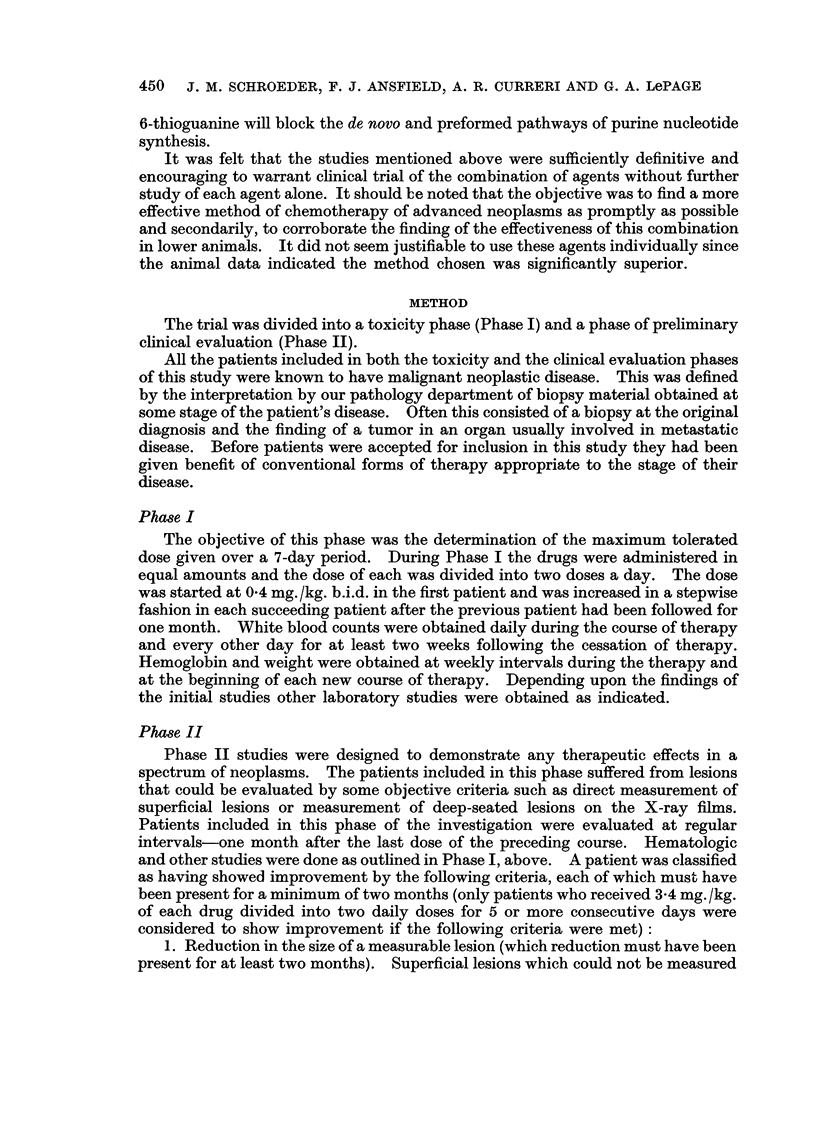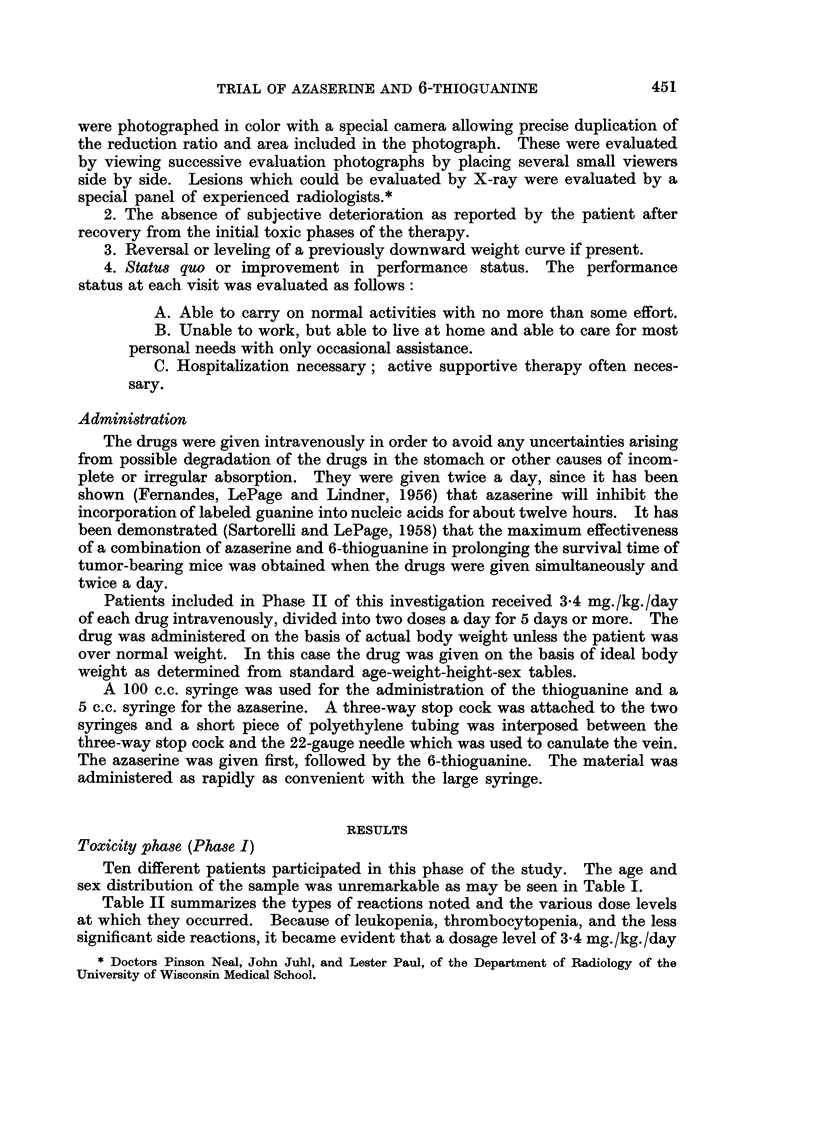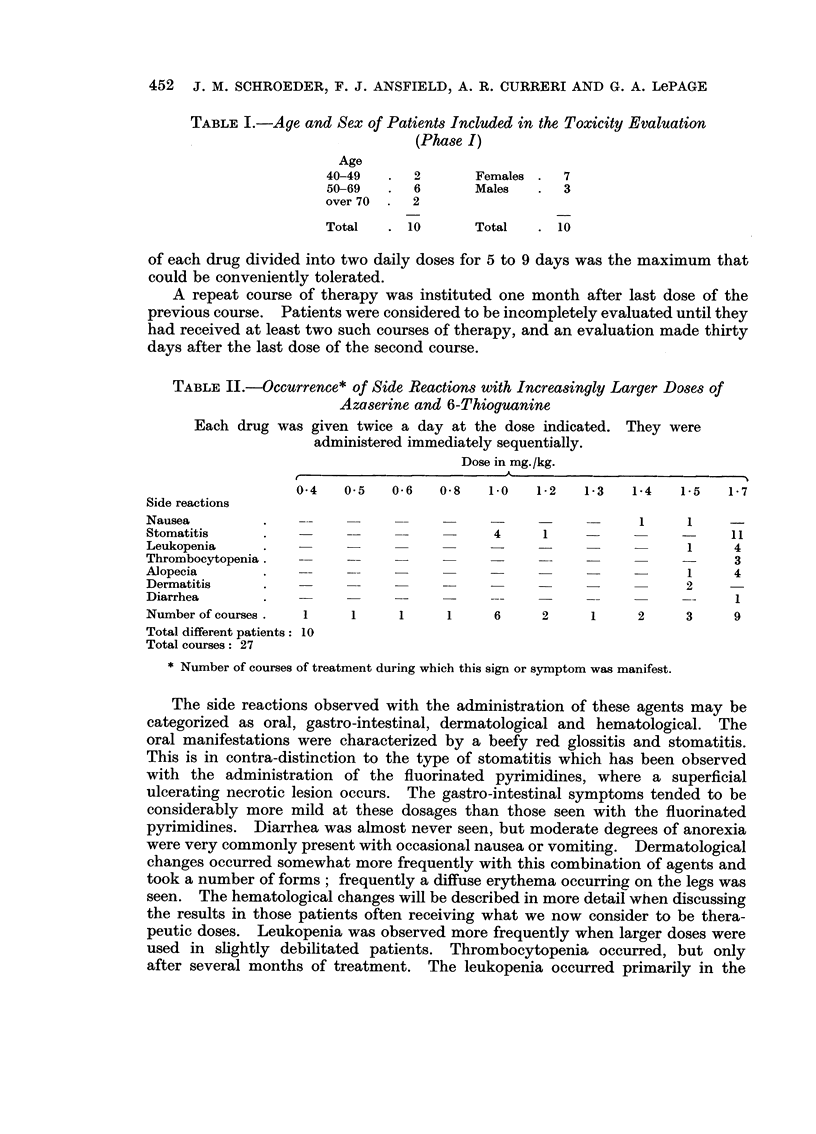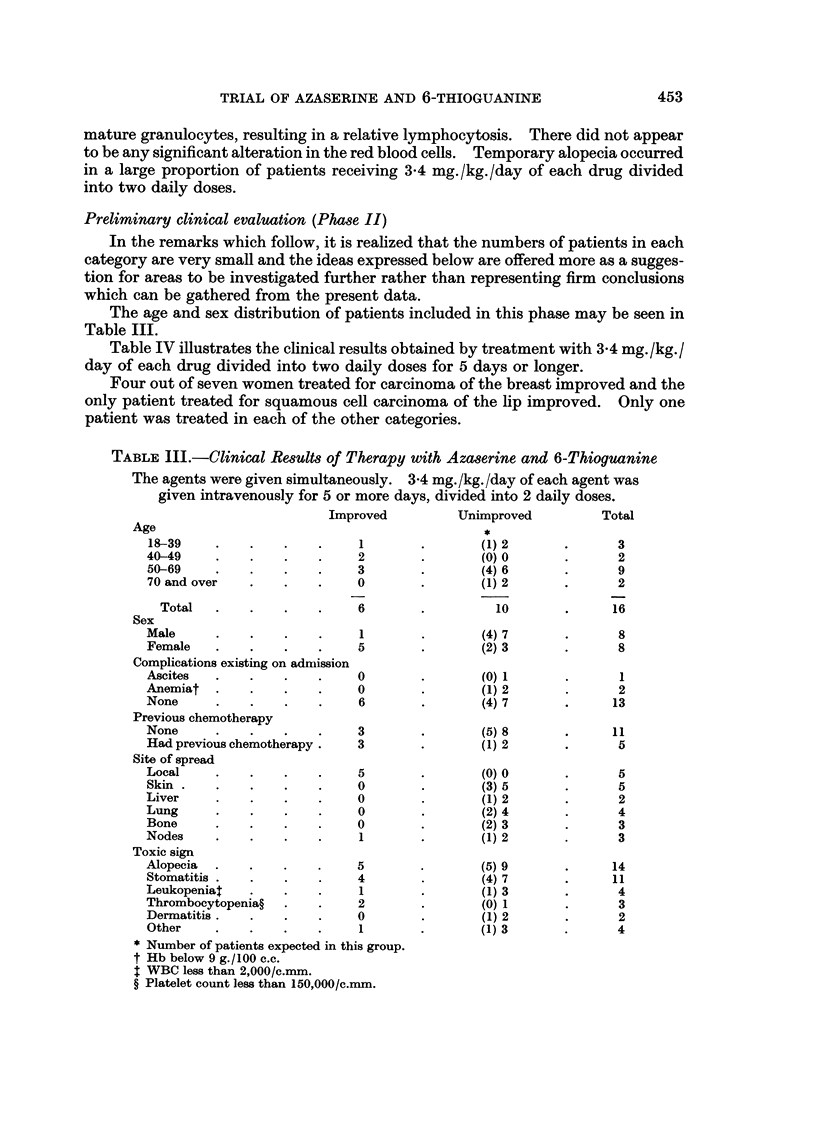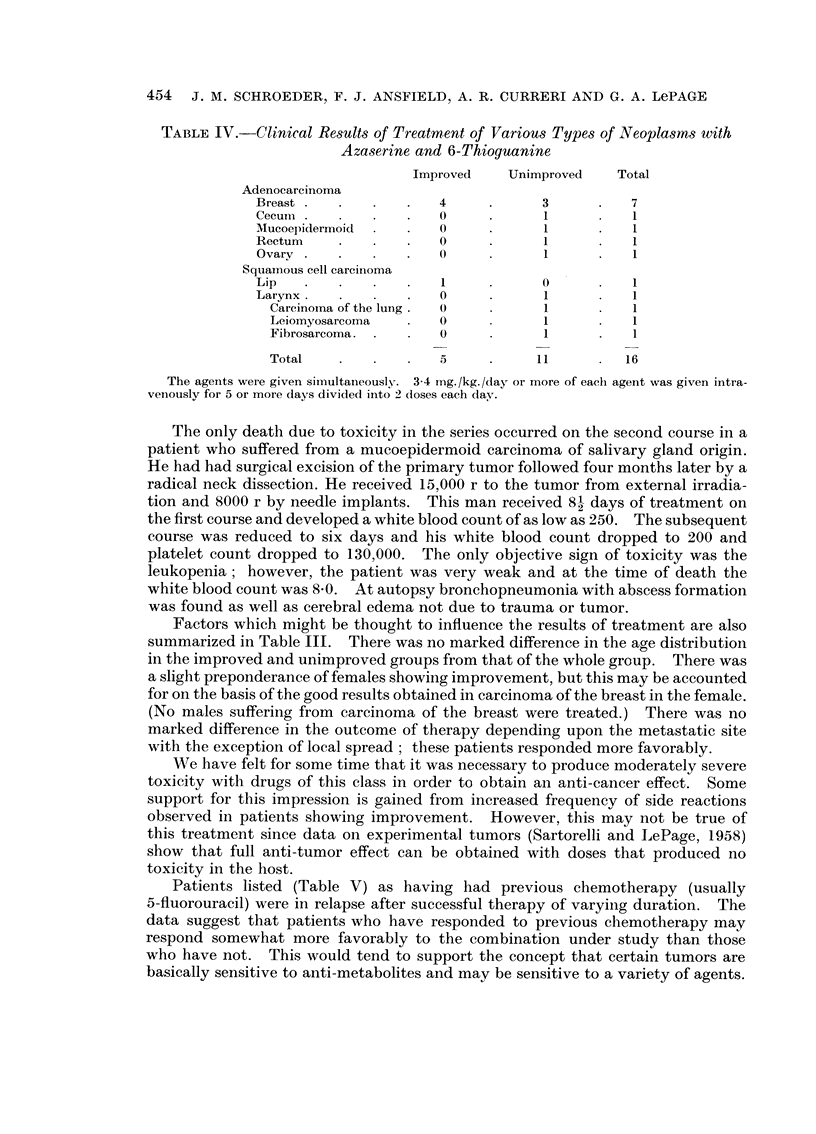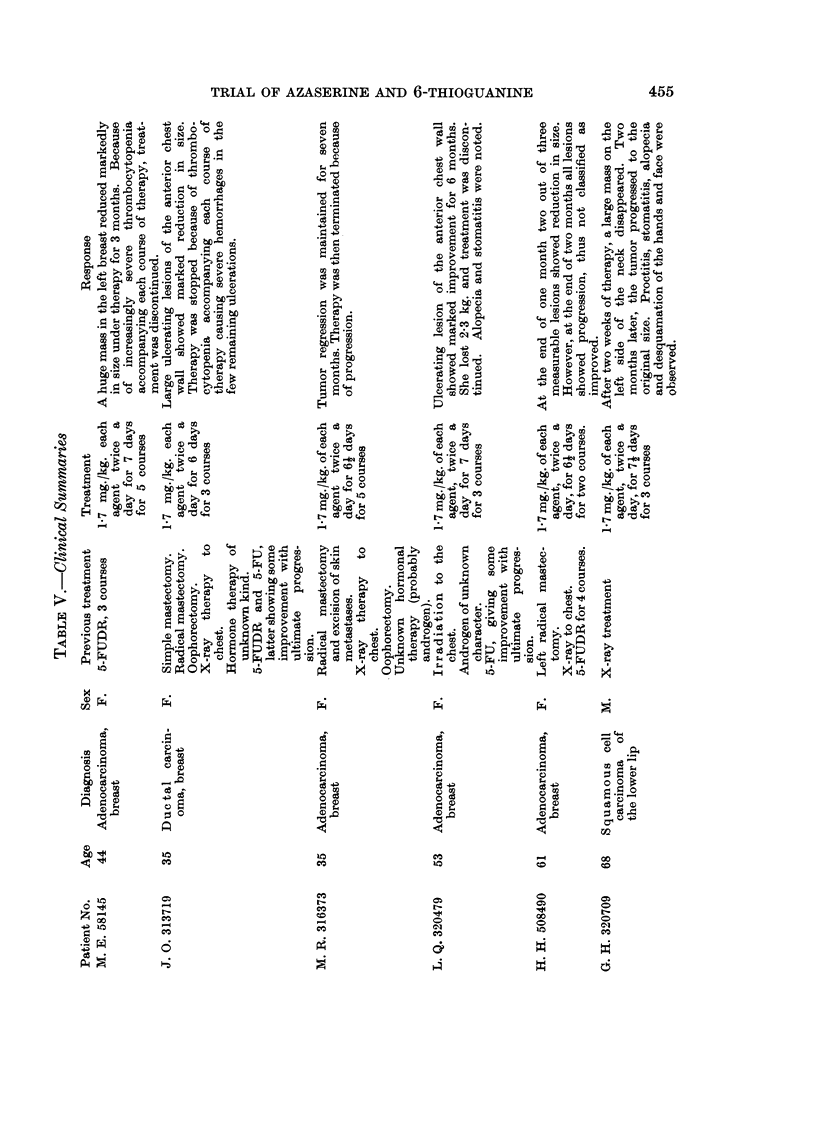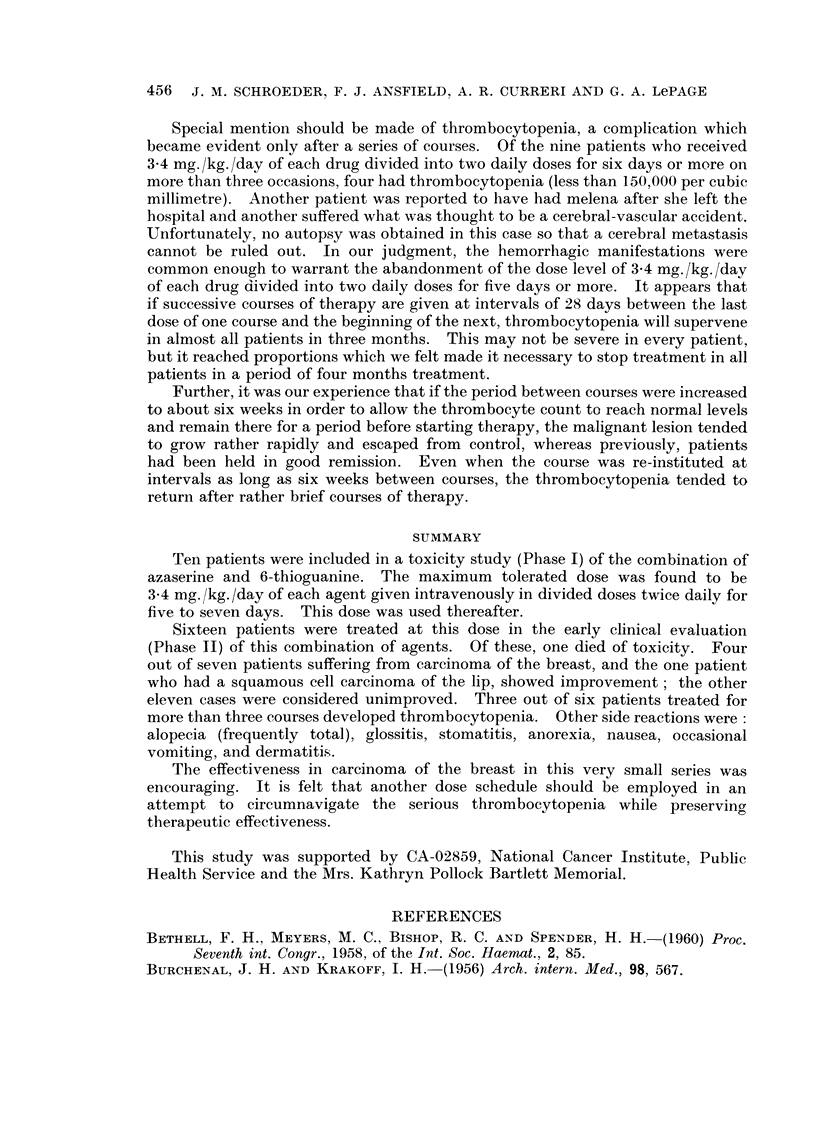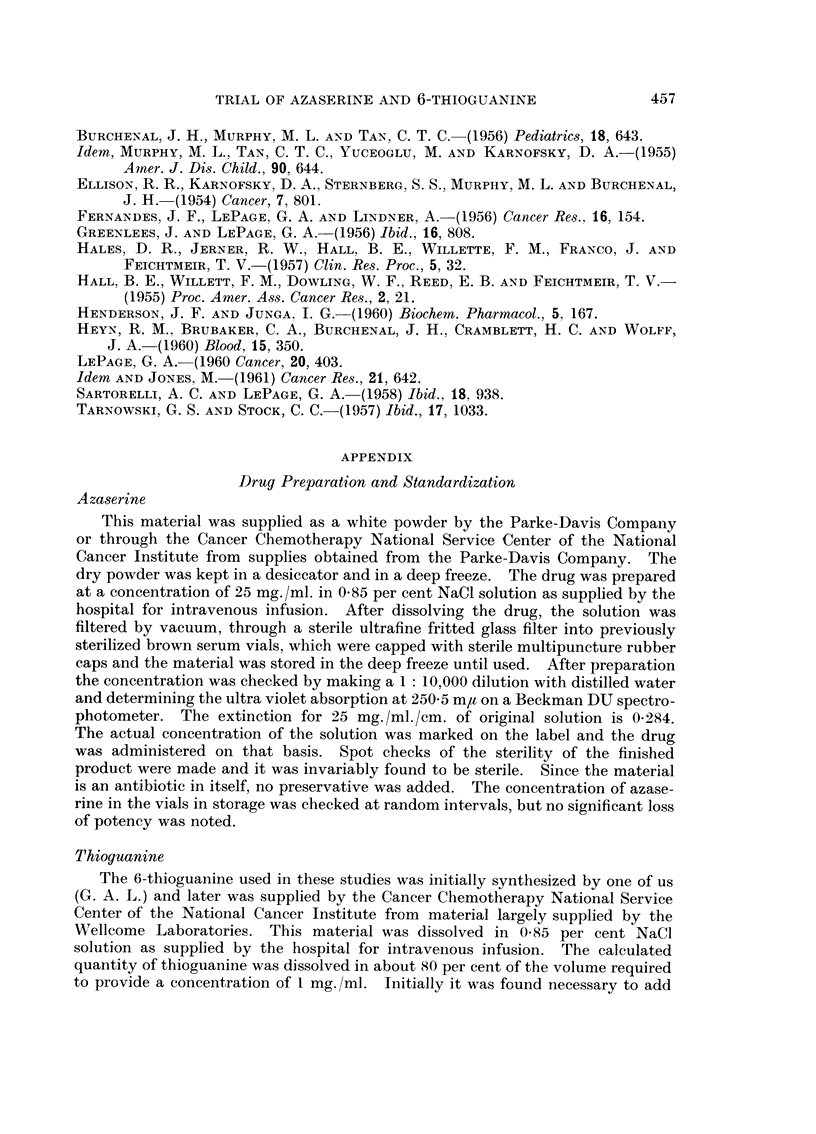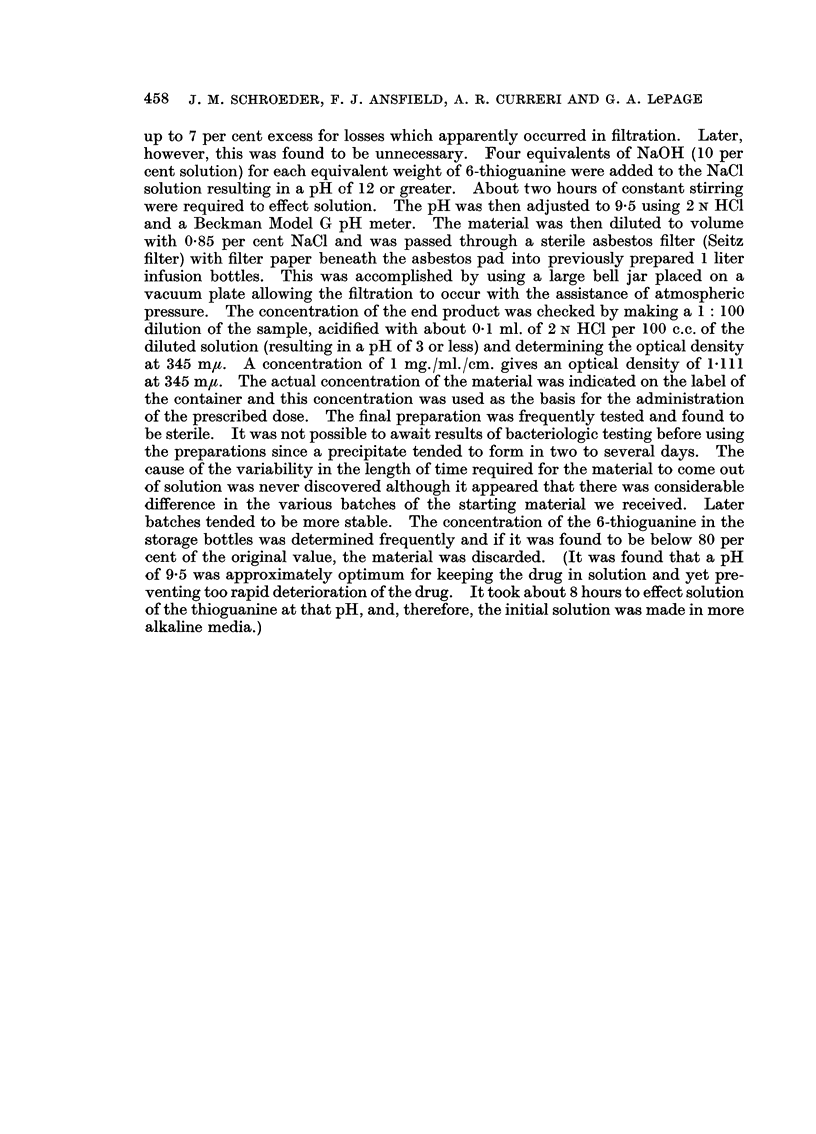# Toxicity and Clinical Trial of Azaserine and 6-Thioguanine in Advanced Solid Malignant Neoplasms

**DOI:** 10.1038/bjc.1964.51

**Published:** 1964-09

**Authors:** J. M. Schroeder, F. J. Ansfield, A. R. Curreri, G. A. LePage


### TOXICITY AND CLINICAL TRIAL OF AZASERINE AND 6-THIOGUANINE IN ADVANCED SOLID MALIGNANT NEOPLASMS

J. M. SCHROEDER,* F. J. ANSFIELD,* A. R. CURRERI,* AND G. A. LEPAGE†

*From the *Cancer Research Hospital, Madison, Wisconsin, and the †Stanford Research Institute, Palo Alto, California, U.S.A.*

Received for publication March 13, 1964

AZASERINE alone was found to be ineffective in a limited spectrum of human solid tumors, except for short term improvement in 5 out of 44 trials in Hodgkin's disease (Ellison *et al.*, 1954). Six-thioguanine used alone in leukemia was found to be somewhat more toxic and less effective than 6-mercaptopurine (Hales *et al.*, 1957).

Somewhat later Bethel *et al.* (1960) found little difference in the effectiveness of 6-mercaptopurine and 6-thioguanine. Some advantage has been found in the combination of azaserine and 6-thioguanine when used in the treatment of mouse mammary carcinoma (Tarnowski and Stock, 1957).

The structurely similar compound, 6-mercaptopurine, has been used in combination with azaserine in the treatment of human leukemias (Burchenal *et al.*, 1955; Hall *et al.*, 1955; Burchenal and Krakoff, 1956; Burchenal, Murphy and Tan, 1956). A careful co-operative evaluation of the combination revealed no advantage over the use of 6-mercaptopurine alone (Heyn *et al.*, 1960).

The mechanism of action of 6-thioguanine is different from that of 6-mercaptopurine (LePage and Jones, 1961). It has been shown that 6-thioguanine is incorporated into nucleic acids of sensitive cell lines (LePage, 1960), while this correlation is absent in the case of 6-mercaptopurine.

In addition, Henderson and Junga (1960) found that a 6-thioguanine resistant subline of Ehrlich carcinoma was not completely resistant to 6-mercaptopurine.

It has been shown that azaserine will cause essentially complete inhibition of the *de novo* purine synthesis in sensitive tumor cells (Greenlees and LePage, 1956). It has been further demonstrated that there is a greater utilization of preformed purines (for the synthesis of essential nucleic acid) in neoplastic cell lines resistant to inhibition by azaserine. Since a compound was being sought to exploit the dependence of certain cell lines on the preformed pathway to purine synthesis made necessary by the blockade of the *de novo* pathway by azaserine, 6-thioguanine seemed to be a desirable choice.

The decision to place the combination of 6-thioguanine and azaserine into clinical trial was based on the encouraging results (Sartorelli and LePage, 1958) obtained by one of us in the treatment of Ehrlich ascites cell tumors in Swiss mice by this combination. These studies indicated that the survival times of animals treated with an appropriate combination of azaserine and 6-thioguanine were markedly prolonged as compared to the survival times of animals treated with either agent alone, even in larger doses. This was accomplished with minimal side effects. It has been shown that concurrent administration of azaserine and

6-thioguanine will block the *de novo* and preformed pathways of purine nucleotide synthesis.

It was felt that the studies mentioned above were sufficiently definitive and encouraging to warrant clinical trial of the combination of agents without further study of each agent alone. It should be noted that the objective was to find a more effective method of chemotherapy of advanced neoplasms as promptly as possible and secondarily, to corroborate the finding of the effectiveness of this combination in lower animals. It did not seem justifiable to use these agents individually since the animal data indicated the method chosen was significantly superior.

### METHOD

The trial was divided into a toxicity phase (Phase I) and a phase of preliminary clinical evaluation (Phase II).

All the patients included in both the toxicity and the clinical evaluation phases of this study were known to have malignant neoplastic disease. This was defined by the interpretation by our pathology department of biopsy material obtained at some stage of the patient's disease. Often this consisted of a biopsy at the original diagnosis and the finding of a tumor in an organ usually involved in metastatic disease. Before patients were accepted for inclusion in this study they had been given benefit of conventional forms of therapy appropriate to the stage of their disease.

### *Phase I*

The objective of this phase was the determination of the maximum tolerated dose given over a 7-day period. During Phase I the drugs were administered in equal amounts and the dose of each was divided into two doses a day. The dose was started at 0·4 mg./kg. b.i.d. in the first patient and was increased in a stepwise fashion in each succeeding patient after the previous patient had been followed for one month. White blood counts were obtained daily during the course of therapy and every other day for at least two weeks following the cessation of therapy. Hemoglobin and weight were obtained at weekly intervals during the therapy and at the beginning of each new course of therapy. Depending upon the findings of the initial studies other laboratory studies were obtained as indicated.

### *Phase II*

Phase II studies were designed to demonstrate any therapeutic effects in a spectrum of neoplasms. The patients included in this phase suffered from lesions that could be evaluated by some objective criteria such as direct measurement of superficial lesions or measurement of deep-seated lesions on the X-ray films. Patients included in this phase of the investigation were evaluated at regular intervals—one month after the last dose of the preceding course. Hematologic and other studies were done as outlined in Phase I, above. A patient was classified as having showed improvement by the following criteria, each of which must have been present for a minimum of two months (only patients who received 3·4 mg./kg. of each drug divided into two daily doses for 5 or more consecutive days were considered to show improvement if the following criteria were met):

1. Reduction in the size of a measurable lesion (which reduction must have been present for at least two months). Superficial lesions which could not be measured were photographed in color with a special camera allowing precise duplication of the reduction ratio and area included in the photograph. These were evaluated by viewing successive evaluation photographs by placing several small viewers side by side. Lesions which could be evaluated by X-ray were evaluated by a special panel of experienced radiologists.*

2. The absence of subjective deterioration as reported by the patient after recovery from the initial toxic phases of the therapy.

3. Reversal or leveling of a previously downward weight curve if present.

4. *Status quo* or improvement in performance status. The performance status at each visit was evaluated as follows :

A. Able to carry on normal activities with no more than some effort.

B. Unable to work, but able to live at home and able to care for most personal needs with only occasional assistance.

C. Hospitalization necessary ; active supportive therapy often necessary.

### *Administration*

The drugs were given intravenously in order to avoid any uncertainties arising from possible degradation of the drugs in the stomach or other causes of incomplete or irregular absorption. They were given twice a day, since it has been shown (Fernandes, LePage and Lindner, 1956) that azaserine will inhibit the incorporation of labeled guanine into nucleic acids for about twelve hours. It has been demonstrated (Sartorelli and LePage, 1958) that the maximum effectiveness of a combination of azaserine and 6-thioguanine in prolonging the survival time of tumor-bearing mice was obtained when the drugs were given simultaneously and twice a day.

Patients included in Phase II of this investigation received 3·4 mg./kg./day of each drug intravenously, divided into two doses a day for 5 days or more. The drug was administered on the basis of actual body weight unless the patient was over normal weight. In this case the drug was given on the basis of ideal body weight as determined from standard age-weight-height-sex tables.

A 100 c.c. syringe was used for the administration of the thioguanine and a 5 c.c. syringe for the azaserine. A three-way stop cock was attached to the two syringes and a short piece of polyethylene tubing was interposed between the three-way stop cock and the 22-gauge needle which was used to canulate the vein. The azaserine was given first, followed by the 6-thioguanine. The material was administered as rapidly as convenient with the large syringe.

### RESULTS

### *Toxicity phase (Phase I)*

Ten different patients participated in this phase of the study. The age and sex distribution of the sample was unremarkable as may be seen in Table I.

Table II summarizes the types of reactions noted and the various dose levels at which they occurred. Because of leukopenia, thrombocytopenia, and the less significant side reactions, it became evident that a dosage level of 3·4 mg./kg./day

* Doctors Pinson Neal, John Juhl, and Lester Paul, of the Department of Radiology of the University of Wisconsin Medical School.

TABLE I.—*Age and Sex of Patients Included in the Toxicity Evaluation (Phase I)*

| Age | | | |
|---|---|---|---|
| 40–49 | 2 | Females | 7 |
| 50–69 | 6 | Males | 3 |
| over 70 | 2 | | |
| Total | 10 | Total | 10 |

of each drug divided into two daily doses for 5 to 9 days was the maximum that could be conveniently tolerated.

A repeat course of therapy was instituted one month after last dose of the previous course. Patients were considered to be incompletely evaluated until they had received at least two such courses of therapy, and an evaluation made thirty days after the last dose of the second course.

TABLE II.—*Occurrence* of Side Reactions with Increasingly Larger Doses of Azaserine and 6-Thioguanine*

Each drug was given twice a day at the dose indicated. They were administered immediately sequentially.

| | Dose in mg./kg. | | | | | | | | | |
|---|---|---|---|---|---|---|---|---|---|---|
| Side reactions | 0·4 | 0·5 | 0·6 | 0·8 | 1·0 | 1·2 | 1·3 | 1·4 | 1·5 | 1·7 |
| Nausea | — | — | — | — | — | — | — | 1 | 1 | — |
| Stomatitis | — | — | — | — | 4 | 1 | — | — | — | 11 |
| Leukopenia | — | — | — | — | — | — | — | — | 1 | 4 |
| Thrombocytopenia | — | — | — | — | — | — | — | — | — | 3 |
| Alopecia | — | — | — | — | — | — | — | — | 1 | 4 |
| Dermatitis | — | — | — | — | — | — | — | — | 2 | — |
| Diarrhea | — | — | — | — | — | — | — | — | — | 1 |
| Number of courses | 1 | 1 | 1 | 1 | 6 | 2 | 1 | 2 | 3 | 9 |

Total different patients : 10
Total courses : 27

* Number of courses of treatment during which this sign or symptom was manifest.

The side reactions observed with the administration of these agents may be categorized as oral, gastro-intestinal, dermatological and hematological. The oral manifestations were characterized by a beefy red glossitis and stomatitis. This is in contra-distinction to the type of stomatitis which has been observed with the administration of the fluorinated pyrimidines, where a superficial ulcerating necrotic lesion occurs. The gastro-intestinal symptoms tended to be considerably more mild at these dosages than those seen with the fluorinated pyrimidines. Diarrhea was almost never seen, but moderate degrees of anorexia were very commonly present with occasional nausea or vomiting. Dermatological changes occurred somewhat more frequently with this combination of agents and took a number of forms ; frequently a diffuse erythema occurring on the legs was seen. The hematological changes will be described in more detail when discussing the results in those patients often receiving what we now consider to be therapeutic doses. Leukopenia was observed more frequently when larger doses were used in slightly debilitated patients. Thrombocytopenia occurred, but only after several months of treatment. The leukopenia occurred primarily in the mature granulocytes, resulting in a relative lymphocytosis. There did not appear to be any significant alteration in the red blood cells. Temporary alopecia occurred in a large proportion of patients receiving 3·4 mg./kg./day of each drug divided into two daily doses.

*Preliminary clinical evaluation (Phase II)*

In the remarks which follow, it is realized that the numbers of patients in each category are very small and the ideas expressed below are offered more as a suggestion for areas to be investigated further rather than representing firm conclusions which can be gathered from the present data.

The age and sex distribution of patients included in this phase may be seen in Table III.

Table IV illustrates the clinical results obtained by treatment with 3·4 mg./kg./day of each drug divided into two daily doses for 5 days or longer.

Four out of seven women treated for carcinoma of the breast improved and the only patient treated for squamous cell carcinoma of the lip improved. Only one patient was treated in each of the other categories.

TABLE III.—*Clinical Results of Therapy with Azaserine and 6-Thioguanine*

The agents were given simultaneously. 3·4 mg./kg./day of each agent was given intravenously for 5 or more days, divided into 2 daily doses.

| | Improved | Unimproved | Total |
|---|---|---|---|
| Age | | * | |
| 18–39 | 1 | (1) 2 | 3 |
| 40–49 | 2 | (0) 0 | 2 |
| 50–69 | 3 | (4) 6 | 9 |
| 70 and over | 0 | (1) 2 | 2 |
| Total | 6 | 10 | 16 |
| Sex | | | |
| Male | 1 | (4) 7 | 8 |
| Female | 5 | (2) 3 | 8 |
| Complications existing on admission | | | |
| Ascites | 0 | (0) 1 | 1 |
| Anemia† | 0 | (1) 2 | 2 |
| None | 6 | (4) 7 | 13 |
| Previous chemotherapy | | | |
| None | 3 | (5) 8 | 11 |
| Had previous chemotherapy | 3 | (1) 2 | 5 |
| Site of spread | | | |
| Local | 5 | (0) 0 | 5 |
| Skin | 0 | (3) 5 | 5 |
| Liver | 0 | (1) 2 | 2 |
| Lung | 0 | (2) 4 | 4 |
| Bone | 0 | (2) 3 | 3 |
| Nodes | 1 | (1) 2 | 3 |
| Toxic sign | | | |
| Alopecia | 5 | (5) 9 | 14 |
| Stomatitis | 4 | (4) 7 | 11 |
| Leukopenia‡ | 1 | (1) 3 | 4 |
| Thrombocytopenia§ | 2 | (0) 1 | 3 |
| Dermatitis | 0 | (1) 2 | 2 |
| Other | 1 | (1) 3 | 4 |

* Number of patients expected in this group.
† Hb below 9 g./100 c.c.
‡ WBC less than 2,000/c.mm.
§ Platelet count less than 150,000/c.mm.

TABLE IV.—*Clinical Results of Treatment of Various Types of Neoplasms with Azaserine and 6-Thioguanine*

| | Improved | Unimproved | Total |
|---|---|---|---|
| Adenocarcinoma | | | |
| Breast | 4 | 3 | 7 |
| Cecum | 0 | 1 | 1 |
| Mucoepidermoid | 0 | 1 | 1 |
| Rectum | 0 | 1 | 1 |
| Ovary | 0 | 1 | 1 |
| Squamous cell carcinoma | | | |
| Lip | 1 | 0 | 1 |
| Larynx | 0 | 1 | 1 |
| Carcinoma of the lung | 0 | 1 | 1 |
| Leiomyosarcoma | 0 | 1 | 1 |
| Fibrosarcoma | 0 | 1 | 1 |
| Total | 5 | 11 | 16 |

The agents were given simultaneously. 3·4 mg./kg./day or more of each agent was given intravenously for 5 or more days divided into 2 doses each day.

The only death due to toxicity in the series occurred on the second course in a patient who suffered from a mucoepidermoid carcinoma of salivary gland origin. He had had surgical excision of the primary tumor followed four months later by a radical neck dissection. He received 15,000 r to the tumor from external irradiation and 8000 r by needle implants. This man received $8\frac{1}{2}$ days of treatment on the first course and developed a white blood count of as low as 250. The subsequent course was reduced to six days and his white blood count dropped to 200 and platelet count dropped to 130,000. The only objective sign of toxicity was the leukopenia ; however, the patient was very weak and at the time of death the white blood count was 8·0. At autopsy bronchopneumonia with abscess formation was found as well as cerebral edema not due to trauma or tumor.

Factors which might be thought to influence the results of treatment are also summarized in Table III. There was no marked difference in the age distribution in the improved and unimproved groups from that of the whole group. There was a slight preponderance of females showing improvement, but this may be accounted for on the basis of the good results obtained in carcinoma of the breast in the female. (No males suffering from carcinoma of the breast were treated.) There was no marked difference in the outcome of therapy depending upon the metastatic site with the exception of local spread ; these patients responded more favorably.

We have felt for some time that it was necessary to produce moderately severe toxicity with drugs of this class in order to obtain an anti-cancer effect. Some support for this impression is gained from increased frequency of side reactions observed in patients showing improvement. However, this may not be true of this treatment since data on experimental tumors (Sartorelli and LePage, 1958) show that full anti-tumor effect can be obtained with doses that produced no toxicity in the host.

Patients listed (Table V) as having had previous chemotherapy (usually 5-fluorouracil) were in relapse after successful therapy of varying duration. The data suggest that patients who have responded to previous chemotherapy may respond somewhat more favorably to the combination under study than those who have not. This would tend to support the concept that certain tumors are basically sensitive to anti-metabolites and may be sensitive to a variety of agents.

TABLE V.—*Clinical Summaries*

| Patient No. | Age | Diagnosis | Sex | Previous treatment | Treatment | Response |
|---|---|---|---|---|---|---|
| M. E. 58145 | 44 | Adenocarcinoma, breast | F. | 5-FUDR, 3 courses | 1·7 mg./kg. each agent twice a day for 7 days for 5 courses | A huge mass in the left breast reduced markedly in size under therapy for 3 months. Because of increasingly severe thrombocytopenia accompanying each course of therapy, treatment was discontinued. |
| J. O. 313719 | 35 | Ductal carcinoma, breast | F. | Simple mastectomy. Radical mastectomy. Oophorectomy. X-ray therapy to chest. Hormone therapy of unknown kind. 5-FUDR and 5-FU, latter showing some improvement with ultimate progression. | 1·7 mg./kg. each agent twice a day for 6 days for 3 courses | Large ulcerating lesions of the anterior chest wall showed marked reduction in size. Therapy was stopped because of thrombocytopenia accompanying each course of therapy causing severe hemorrhages in the few remaining ulcerations. |
| M. R. 316373 | 35 | Adenocarcinoma, breast | F. | Radical mastectomy and excision of skin metastases. X-ray therapy to chest. Oophorectomy. Unknown hormonal therapy (probably androgen). | 1·7 mg./kg. of each agent twice a day for 6½ days for 5 courses | Tumor regression was maintained for seven months. Therapy was then terminated because of progression. |
| L. Q. 320479 | 53 | Adenocarcinoma, breast | F. | Irradiation to the chest. Androgen of unknown character. 5-FU, giving some improvement with ultimate progression. | 1·7 mg./kg. of each agent, twice a day for 7 days for 3 courses | Ulcerating lesion of the anterior chest wall showed marked improvement for 6 months. She lost 2·3 kg. and treatment was discontinued. Alopecia and stomatitis were noted. |
| H. H. 508490 | 61 | Adenocarcinoma, breast | F. | Left radical mastectomy. X-ray to chest. 5-FUDR for 4 courses. | 1·7 mg./kg. of each agent, twice a day, for 6½ days for two courses. | At the end of one month two out of three measurable lesions showed reduction in size. However, at the end of two months all lesions showed progression, thus not classified as improved. |
| G. H. 320709 | 68 | Squamous cell carcinoma of the lower lip | M. | X-ray treatment | 1·7 mg./kg. of each agent, twice a day, for 7½ days for 3 courses | After two weeks of therapy, a large mass on the left side of the neck disappeared. Two months later, the tumor progressed to the original size. Proctitis, stomatitis, alopecia and desquamation of the hands and face were observed. |

Special mention should be made of thrombocytopenia, a complication which became evident only after a series of courses. Of the nine patients who received 3·4 mg./kg./day of each drug divided into two daily doses for six days or more on more than three occasions, four had thrombocytopenia (less than 150,000 per cubic millimetre). Another patient was reported to have had melena after she left the hospital and another suffered what was thought to be a cerebral-vascular accident. Unfortunately, no autopsy was obtained in this case so that a cerebral metastasis cannot be ruled out. In our judgment, the hemorrhagic manifestations were common enough to warrant the abandonment of the dose level of 3·4 mg./kg./day of each drug divided into two daily doses for five days or more. It appears that if successive courses of therapy are given at intervals of 28 days between the last dose of one course and the beginning of the next, thrombocytopenia will supervene in almost all patients in three months. This may not be severe in every patient, but it reached proportions which we felt made it necessary to stop treatment in all patients in a period of four months treatment.

Further, it was our experience that if the period between courses were increased to about six weeks in order to allow the thrombocyte count to reach normal levels and remain there for a period before starting therapy, the malignant lesion tended to grow rather rapidly and escaped from control, whereas previously, patients had been held in good remission. Even when the course was re-instituted at intervals as long as six weeks between courses, the thrombocytopenia tended to return after rather brief courses of therapy.

### SUMMARY

Ten patients were included in a toxicity study (Phase I) of the combination of azaserine and 6-thioguanine. The maximum tolerated dose was found to be 3·4 mg./kg./day of each agent given intravenously in divided doses twice daily for five to seven days. This dose was used thereafter.

Sixteen patients were treated at this dose in the early clinical evaluation (Phase II) of this combination of agents. Of these, one died of toxicity. Four out of seven patients suffering from carcinoma of the breast, and the one patient who had a squamous cell carcinoma of the lip, showed improvement ; the other eleven cases were considered unimproved. Three out of six patients treated for more than three courses developed thrombocytopenia. Other side reactions were : alopecia (frequently total), glossitis, stomatitis, anorexia, nausea, occasional vomiting, and dermatitis.

The effectiveness in carcinoma of the breast in this very small series was encouraging. It is felt that another dose schedule should be employed in an attempt to circumnavigate the serious thrombocytopenia while preserving therapeutic effectiveness.

This study was supported by CA-02859, National Cancer Institute, Public Health Service and the Mrs. Kathryn Pollock Bartlett Memorial.

### REFERENCES

BETHELL, F. H., MEYERS, M. C., BISHOP, R. C. AND SPENDER, H. H.—(1960) *Proc. Seventh int. Congr.*, 1958, of the *Int. Soc. Haemat.*, **2**, 85.

BURCHENAL, J. H. AND KRAKOFF, I. H.—(1956) *Arch. intern. Med.*, **98**, 567.

BURCHENAL, J. H., MURPHY, M. L. AND TAN, C. T. C.—(1956) *Pediatrics*, **18**, 643.
*Idem*, MURPHY, M. L., TAN, C. T. C., YUCEOGLU, M. AND KARNOFSKY, D. A.—(1955) *Amer. J. Dis. Child.*, **90**, 644.
ELLISON, R. R., KARNOFSKY, D. A., STERNBERG, S. S., MURPHY, M. L. AND BURCHENAL, J. H.—(1954) *Cancer*, **7**, 801.
FERNANDES, J. F., LEPAGE, G. A. AND LINDNER, A.—(1956) *Cancer Res.*, **16**, 154.
GREENLEES, J. AND LEPAGE, G. A.—(1956) *Ibid.*, **16**, 808.
HALES, D. R., JERNER, R. W., HALL, B. E., WILLETTE, F. M., FRANCO, J. AND FEICHTMEIR, T. V.—(1957) *Clin. Res. Proc.*, **5**, 32.
HALL, B. E., WILLETT, F. M., DOWLING, W. F., REED, E. B. AND FEICHTMEIR, T. V.—(1955) *Proc. Amer. Ass. Cancer Res.*, **2**, 21.
HENDERSON, J. F. AND JUNGA, I. G.—(1960) *Biochem. Pharmacol.*, **5**, 167.
HEYN, R. M., BRUBAKER, C. A., BURCHENAL, J. H., CRAMBLETT, H. C. AND WOLFF, J. A.—(1960) *Blood*, **15**, 350.
LEPAGE, G. A.—(1960 *Cancer*, **20**, 403.
*Idem* AND JONES, M.—(1961) *Cancer Res.*, **21**, 642.
SARTORELLI, A. C. AND LEPAGE, G. A.—(1958) *Ibid.*, **18**, 938.
TARNOWSKI, G. S. AND STOCK, C. C.—(1957) *Ibid.*, **17**, 1033.

### APPENDIX

### *Drug Preparation and Standardization*

*Azaserine*

This material was supplied as a white powder by the Parke-Davis Company or through the Cancer Chemotherapy National Service Center of the National Cancer Institute from supplies obtained from the Parke-Davis Company. The dry powder was kept in a desiccator and in a deep freeze. The drug was prepared at a concentration of 25 mg./ml. in 0·85 per cent NaCl solution as supplied by the hospital for intravenous infusion. After dissolving the drug, the solution was filtered by vacuum, through a sterile ultrafine fritted glass filter into previously sterilized brown serum vials, which were capped with sterile multipuncture rubber caps and the material was stored in the deep freeze until used. After preparation the concentration was checked by making a 1 : 10,000 dilution with distilled water and determining the ultra violet absorption at 250·5 m$\mu$ on a Beckman DU spectrophotometer. The extinction for 25 mg./ml./cm. of original solution is 0·284. The actual concentration of the solution was marked on the label and the drug was administered on that basis. Spot checks of the sterility of the finished product were made and it was invariably found to be sterile. Since the material is an antibiotic in itself, no preservative was added. The concentration of azaserine in the vials in storage was checked at random intervals, but no significant loss of potency was noted.

*Thioguanine*

The 6-thioguanine used in these studies was initially synthesized by one of us (G. A. L.) and later was supplied by the Cancer Chemotherapy National Service Center of the National Cancer Institute from material largely supplied by the Wellcome Laboratories. This material was dissolved in 0·85 per cent NaCl solution as supplied by the hospital for intravenous infusion. The calculated quantity of thioguanine was dissolved in about 80 per cent of the volume required to provide a concentration of 1 mg./ml. Initially it was found necessary to add up to 7 per cent excess for losses which apparently occurred in filtration. Later, however, this was found to be unnecessary. Four equivalents of NaOH (10 per cent solution) for each equivalent weight of 6-thioguanine were added to the NaCl solution resulting in a pH of 12 or greater. About two hours of constant stirring were required to effect solution. The pH was then adjusted to 9·5 using 2 N HCl and a Beckman Model G pH meter. The material was then diluted to volume with 0·85 per cent NaCl and was passed through a sterile asbestos filter (Seitz filter) with filter paper beneath the asbestos pad into previously prepared 1 liter infusion bottles. This was accomplished by using a large bell jar placed on a vacuum plate allowing the filtration to occur with the assistance of atmospheric pressure. The concentration of the end product was checked by making a 1 : 100 dilution of the sample, acidified with about 0·1 ml. of 2 N HCl per 100 c.c. of the diluted solution (resulting in a pH of 3 or less) and determining the optical density at 345 m$\mu$. A concentration of 1 mg./ml./cm. gives an optical density of 1·111 at 345 m$\mu$. The actual concentration of the material was indicated on the label of the container and this concentration was used as the basis for the administration of the prescribed dose. The final preparation was frequently tested and found to be sterile. It was not possible to await results of bacteriologic testing before using the preparations since a precipitate tended to form in two to several days. The cause of the variability in the length of time required for the material to come out of solution was never discovered although it appeared that there was considerable difference in the various batches of the starting material we received. Later batches tended to be more stable. The concentration of the 6-thioguanine in the storage bottles was determined frequently and if it was found to be below 80 per cent of the original value, the material was discarded. (It was found that a pH of 9·5 was approximately optimum for keeping the drug in solution and yet preventing too rapid deterioration of the drug. It took about 8 hours to effect solution of the thioguanine at that pH, and, therefore, the initial solution was made in more alkaline media.)